# Effect of Anti-IL17 Antibody Treatment Alone and in Combination With Rho-Kinase Inhibitor in a Murine Model of Asthma

**DOI:** 10.3389/fphys.2018.01183

**Published:** 2018-09-05

**Authors:** Tabata M. dos Santos, Renato F. Righetti, Leandro do N. Camargo, Beatriz M. Saraiva-Romanholo, Luciana R. C. R. B. Aristoteles, Flávia C. R. de Souza, Silvia Fukuzaki, Maria I. C. Alonso-Vale, Maysa M. Cruz, Carla M. Prado, Edna A. Leick, Milton A. Martins, Iolanda F. L. C. Tibério

**Affiliations:** ^1^Department of Medicine, Faculdade de Medicina (FMUSP), Universidade de São Paulo, São Paulo, Brazil; ^2^Department of Medicine, Laboratory of Experimental Therapeutics, LIM-20, School of Medicine, University of São Paulo, São Paulo, Brazil; ^3^Department of Medicine, University City of São Paulo (UNICID), São Paulo, Brazil; ^4^Department of Biological Sciences, Federal University of São Paulo, Diadema, Brazil; ^5^Department of Biosciences, Federal University of São Paulo (UNIFESP), Santos, Brazil

**Keywords:** asthma, interleukin-17, Rho-kinase, Y-27632, inflammation, neutralizing antibody

## Abstract

**Background:** Interleukin-17 (IL-17) and Rho-kinase (ROCK) play an important role in regulating the expression of inflammatory mediators, immune cell recruitment, hyper-responsiveness, tissue remodeling, and oxidative stress. Modulation of IL-17 and ROCK proteins may represent a promising approach for the treatment of this disease.

**Objective:** To study the effects of an anti-IL17 neutralizing antibody and ROCK inhibitor treatments, separately and in combination, in a murine model of chronic allergy-induced lung inflammation.

**Methods:** Sixty-four BALBc mice, were divided into eight groups (*n* = 8): SAL (saline-instilled); OVA (exposed-ovalbumin); SAL-RHOi (saline and ROCK inhibitor), OVA-RHOi (exposed-ovalbumin and ROCK inhibitor); SAL-anti-IL17 (saline and anti-IL17); OVA-anti-IL17 (exposed-ovalbumin and anti-IL17); SAL-RHOi-anti-IL17 (saline, ROCK inhibitor and anti-IL17); and OVA-RHOi-anti-IL17 (exposed-ovalbumin, anti-IL17, and ROCK inhibitor). A 28-day protocol of albumin treatment was used for sensitization and induction of pulmonary inflammation. The anti-IL17A neutralizing antibody (7.5 μg per treatment) was administered by intraperitoneal injection and ROCK inhibitor (Y-27632) intranasally (10 mg/kg), 1 h prior to each ovalbumin challenge (days 22, 24, 26, and 28).

**Results:** Treatment with the anti-IL17 neutralizing antibody and ROCK inhibitor attenuated the percentage of maximal increase of respiratory system resistance and respiratory system elastance after challenge with methacholine and the inflammatory response markers evaluated (CD4^+^, CD8^+^, ROCK1, ROCK2, IL-4, IL-5, IL-6, IL-10 IL-13, IL-17, TNF-α, TGF-β, NF-κB, dendritic cells, iNOS, MMP-9, MMP-12, TIMP-1, FOXP3, isoprostane, biglycan, decorin, fibronectin, collagen fibers content and gene expression of IL-17, VAChT, and arginase) compared to the OVA group (*p* < 0.05). Treatment with anti-IL17 and the ROCK inhibitor together resulted in potentiation in decreasing the percentage of resistance increase after challenge with methacholine, decreased the number of IL-5 positive cells in the airway, and reduced, IL-5, TGF-β, FOXP3, ROCK1 and ROCK2 positive cells in the alveolar septa compared to the OVA-RHOi and OVA-anti-IL17 groups (*p* < 0.05).

**Conclusion:** Anti-IL17 treatment alone or in conjunction with the ROCK inhibitor, modulates airway responsiveness, inflammation, tissue remodeling, and oxidative stress in mice with chronic allergic lung inflammation.

## Introduction

Asthma is a chronic airway disease characterized by bronchial hyper-responsiveness, lung inflammation, airway remodeling, and airway obstruction (Meyer et al., 2014; Rampadarath and Donovan, 2018). As heterogeneous condition, asthma includes acute episodes that revert spontaneously or with treatment and chronic inflammation and/or structural changes that may be related to persistent symptoms and eventual reduction of pulmonary function. Personalized treatment is often needed to control severe asthma. These patients require constant attention to avoid exacerbating the disease and the associated unnecessary costs to health and quality of life (Reddel et al., 2009; Lemanske and Busse, 2010; Wang et al., 2018).

Sensitization is the initial phase of asthma in which dendritic cells present in the bronchial mucosa orchestrate an immune response upon antigen exposure (Hamid et al., 2003; Leffler et al., 2018). The release of pro inflammatory mediators (e.g., histamine, reactive oxygen species) trigger an inflammatory response in which mast cells, basophils, eosinophils, and CD4^+^ Th2 lymphocytes help to induce the signs and symptoms characteristic of full-blown asthma (bronchial hyper-reactivity) (Platts-Mills et al., 2001). Th17 profile cells are involved in inflammation. In mice their maturation is induced by activated T cells in the presence of TGF-β and IL-6 (Newcomb et al., 2012). Th17 cells are involved in the inflammatory process in severe asthma and their inhibition interferes with T helper cell mediated inflammation (Camargo et al., 2018). Increased expression of IL-17A has been documented in the lungs, sputum, BALF, and serum of patients with asthma. This cytokine stimulates a Th2 cell response, airway hyper-reactivity, neutrophil infiltration, and mucus production (Doe et al., 2010; Choy et al., 2015; Chenuet et al., 2017). In addition, asthma severity has been shown to positively correlate with IL-17 expression levels (Molet et al., 2001).

Rho-kinase also contributes to the pathogenesis of asthma and also diseases associated with increased smooth muscle tonus or hyper-reactivity, as it is involved in muscle contraction. This protein affects many biological functions phagocytosis, proliferation, secretion, and maintenance of cell morphology (Wettschureck and Offermanns, 2002). In addition, ROCKs participate in cell migration, including T lymphocytes and eosinophils. The RhoA-ROCK-myosin II signaling pathway allows the high-speed invasion of lung infiltrating T cells (Mrass et al., 2017). ROCK may induce changes in the smooth muscle cells (Wei et al., 2014).

Increased intracellular calcium concentration in smooth muscle cells induces contraction. When bound to calcium, the serine–threonine kinase calmodulin directly phosphorylates and thus activates myosin light chain kinase generating muscle contraction; through another pathway, myosin phosphorylation is increased when ROCKs directly phosphorylate light chain phosphatase of the myosin, thereby inactivating the phosphatase (Erle and Sheppard, 2014). Pharmacological inhibitors of ROCK, such as Y-27632 and Fasudil, block its activity and prevent the inhibition of RhoA bound myosin phosphatase, resulting in the relaxation of smooth muscle (Chiba et al., 2010). Recently, this specific inhibition has been associated with reduced recruitment of eosinophils into the airways and pulmonary parenchyma in guinea pigs with chronic pulmonary allergic inflammation, as well as contributing to the attenuation of the remodeling process, demonstrated by lower collagen and elastic fibers content in the walls of the airways and lung tissue (Possa et al., 2012; Righetti et al., 2014).

IL-17A increases the contractile responses in the airway smooth muscle cells, through activation of NF-κB and the subsequent induction of RhoA and ROCK2 expression, which results in increased phosphorylation of the myosin light chain (Kudo et al., 2012). Therefore, our work aimed to further the effective treatment and management of asthma through a ROCK inhibitor alone or in combination with an anti-IL17 neutralizing antibody. This personalized approach should pave the way for combinatorial approaches in the treatment of asthma to control bronchoconstriction, hyper-reactivity, and tissue remodeling.

## Materials and Methods

### Animals

This protocol was approved by the Ethics Committee on the Use of Animals of the School of Medicine of the University of São Paulo, São Paulo, Brazil (Protocol No. 064/15).

This study involved a total of 64 animals and was performed according to the Guidelines for the Care and Use of Laboratory Animals published by the National Institute of Health. For the study, male BALBc mice (20–25 g) were used in an animal facility at the Faculty of Medicine of the University of São Paulo (Schaafsma et al., 2004).

The mice were divided into eight groups of eight animals each:

(1) SAL: inhalation and intraperitoneal injection with saline solution;
(2) OVA: inhalation and intraperitoneal injection with ovalbumin;
(3) SAL-RHOi: inhalation and intraperitoneal injection with saline solution and intranasal treatment with ROCK inhibitor;
(4) OVA-RHOi: inhalation and intraperitoneal injection with ovalbumin and intranasal treatment with ROCK inhibitor;
(5) SAL-anti-IL17: inhalations and intraperitoneal injection with saline solution and intraperitoneal treatment with anti-IL17 monoclonal antibody;
(6) OVA-anti-IL17: inhalation and intraperitoneal injection with ovalbumin and intraperitoneal treatment with anti-IL17 monoclonal antibody;
(7) SAL-RHOi-anti-IL17: inhalation and intraperitoneal injection with saline solution and intranasal treatment with ROCK inhibitor combined with intraperitoneal treatment with anti-IL17 monoclonal antibody;
(8) OVA-RHOi-anti-IL17: inhalation and intraperitoneal injection with ovalbumin and intranasal treatment with ROCK inhibitor combined with intraperitoneal treatment with monoclonal antibody anti-IL17.

### Sensitization Protocol

**FIGURE 1:**
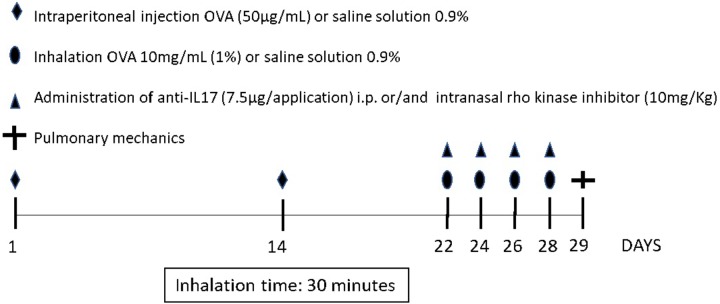
Protocol for sensitization and induction of the inflammatory response. On days 1 and 14, the OVA, OVA-RHOi, OVA-anti-IL17, and OVA-RHOi-anti-IL17 groups were sensitized with ovalbumin (i.p.) and the control groups SAL, SAL-RHOi, SAL-anti-IL17, and SAL-RHOi-anti-IL17 received saline solution (i.p.). On days 22, 24, 26, and 28, 1 h prior to challenge, treatment groups received anti-IL17 (i.p.) and/or ROCK inhibitor (intranasal).

**Figure 1** depicts the protocol for sensitization and induction of pulmonary inflammation from exposure to ovalbumin for 28 days. The animals in the OVA, OVA-RHOi, OVA-RHOi-anti-IL17, and OVA-RHOi-anti-IL17 groups received intraperitoneal injection (i.p.) of 50 μg ovalbumin (GRADE IV, Sigma-Aldrich, St. Louis, MO, United States) and 6 mg aluminum hydroxide (Alumen, Pepsamar, Sanofi-Synthelabo SA, Rio de Janeiro, Brazil) 0.2 mL on days 1 and 14 of the experimental protocol. On days 22, 24, 26, and 28, the animals from the OVA, OVA-RHOi, OVA-anti-IL17, and OVA-RHOi-anti-IL17 groups were placed in an acrylic box (30 cm × 15 cm × 20 cm) with a coupled ultrasonic nebulizer and subjected to aerosol inhalation of ovalbumin (10 mg/mL, 1%) in 0.9% NaCl (saline solution) for 30 min.

On days 1 and 14 of the experimental protocol, animals of the control groups (SAL, SAL-RHOi, SAL-anti-IL17, and SAL-RHOi-anti-IL17) received saline solution (0.9% NaCl) with 6 mg aluminum hydroxide i.p. and exposed on days 22, 24, 26, and 28 to aerosol saline solution for 30 min (Arantes-Costa et al., 2008; Camargo et al., 2018).

### Treatment of Inhibitor Rho-Kinase

SAL-RHOi and OVA-RHOi groups received intranasal treatment with Y-27632 (10 mg/kg), a highly selective ROCK inhibitor, (+) -(R)-*trans*-4-(1-aminoethyl)-*N*-(4-pyridyl) cyclohexanecarboxamide dihydrochloride, monohydrate, 1 h prior to aerosol inhalation on days 22, 24, 26, and 28, as previously described (Henry et al., 2005). Henry et al. (2005) showed that the dose of 3 mg/kg of Y-27632 did not significantly reduce the numbers of BALF eosinophils recovered from allergic mice 96 h postchallenge. Nevertheless, the largest reduction of eosinophils in BALF was produced by the treatment with 10 mg/kg Y-27632. Therefore, we chose the dose of 10 mg/kg (Henry et al., 2005).

### Treatment of Anti-IL-17A

For the dosage of anti-IL-17A neutralizing antibody we based our decision on the study of Barlow et al. (2011) and Camargo et al. (2018). The SAL-anti-IL17 and OVA-anti-IL17 groups received the 7.5 μg of the anti-IL17 neutralizing antibody (i.p.) 1 h prior to aerosol inhalation on days 22, 24, 26, and 28, as previously described. Previous studies demonstrated that this dose decreased inflammation, remodeling, and oxidative stress in animal model of asthma (Barlow et al., 2011; Camargo et al., 2018).

### Treatment With Association of Inhibitor Rho-Kinase and Anti-IL-17A

SAL-RHOi-anti-IL17 and OVA-RHOi-anti-IL17 groups received intranasal treatment with Y-27632 combined with the anti-IL17 neutralizing antibody i.p. 1 h prior to aerosol inhalation on days 22, 24, 26, and 28.

### Evaluation of Pulmonary Mechanics

One day after the termination of the experimental protocol, the animals were anesthetized with thiopental (50 mg/kg i.p.) and tracheostomized. For mechanical ventilation, a tracheostomy was performed by inserting a plastic cannula into the trachea. The animals were connected to a Harvard 683 ventilator (Harvard Apparatus, South Natick, MA, United States) and adjusted parameters with a tidal volume of 10 ml/kg, a respiratory rate of 120 cycles/min and inspiratory flow sine curve. To abolish their ventilatory effort, animals received pancuronium (0.2 mg/kg i.p.) (Righetti et al., 2014).

Tracheal pressure signals and volumes were acquired through differential pressure transducers (Honeywell 163PC01D36, Freeport, IL, United States) and converted by an analog digital board (DT01EZ, Data Translation, Marlboro, MA, United States). The resistance and elastance values of the respiratory system were calculated through the equation of respiratory system movement, described below: Ptr (time) = Rrs.*V*′ (time) + Ers.*V* (*t*), where: Ptr is tracheal pressure, Rrs is resistance, Ers is elastance, *V*′ is airflow, *V* is lung volume, and *t* is time (Righetti et al., 2014).

During mechanical ventilation, the basal measures of resistance and elastance of the animals were performed after 30 s of ventilation. The challenge was performed with inhalation of methacholine at the doses of 3, 30, 300 mg/ml, in the first 30 s, first, second, and third minutes and the measures of resistance and elastance of the respiratory system were obtained. The maximum response of resistance (%Rrs) and elastance (%Ers) of the respiratory system were considered for the study (Righetti et al., 2014; Camargo et al., 2018).

### Bronchoalveolar Lavage

After the respiratory mechanics were evaluated, bronchoalveolar lavage was performed. Saline solution (0.5 mL each) was instilled three times with a syringe through the tracheostomy cannula and a total volume of 1.5 ml was recovered. The BALF was centrifuged at 790 × *g* for 10 min at 5°C with an average mean recovery of 80%.

The cell pellet was resuspended in 300 μL of saline using a vortex. Then, 100 μL was used to prepare a slide for differential cell counting. The remaining BALF was centrifuged onto a slide in the cytospin for 6 min at 450 rpm and then stained with diff quick. Total cell counts were performed by light microscopy with the Neubauer hemocytometer (400×). Differential cell count of eosinophils, macrophages, and neutrophils was performed using an optical light microscope at 1,000× magnification (Saraiva-Romanholo et al., 2003).

### Immunohistochemistry

Tissues were maintained in 4% formaldehyde and embedded in paraffin blocks. The lungs were sectioned longitudinally into three parts with a thickness of 4 μm, contemplating the upper, middle, and lower pulmonary lobes. For a coloring of Picro-Sirius (collagen fibers), cuts were stained for 1 h in Picro-Sirius at room temperature and then washed under running water for 5 min. After this, the sections were stained with Harris hematoxylin for 6 min and later washed under running water for 10 min.

**FIGURE 2:**
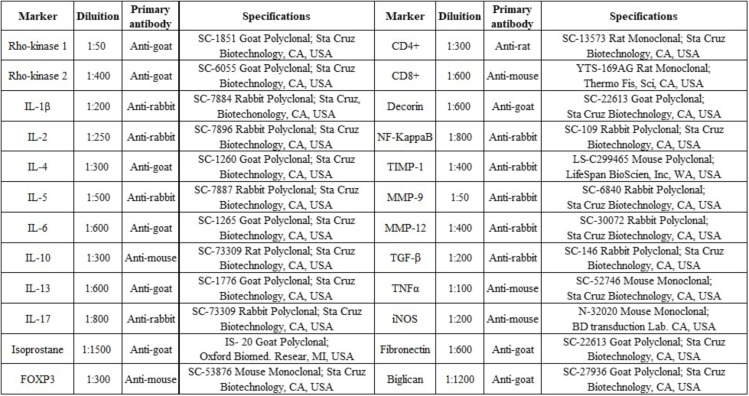
Markers, dilution, primary antibody, and specifications.

To mark the samples, the procedures were performed in the following sequence: antigen retrieval, blocking, and primary antibody incubation (**Figure 2**), secondary antibody incubation, staining, and counterstaining. First dewaxing was performed, followed by hydration, digestion, and recovery of the antigen at high temperature in the steam pan for 50 min (ILs) or in the pressure cooker for 1 min (other cytokines) using citrate pH 6 buffers after this step, peroxidase blocking was performed using of hydrogen peroxide (3%) for 5 min and then washed three times with PBS. The diluted antibodies were pipetted onto the slices and the slides were incubated in a humidity chamber overnight (18–20 h).

After this incubation at 4°C, the slides were washed with PBS and then incubated in the humidity chamber with secondary antibodies (rabbit, rat, goat or mouse) for 30 min at 37°C. After having been washed three times for 3 min with PBS, the slides were incubated in a humidity chamber with the Vector AB Conjugate Complex for 30 min at 37°C, washed again with PBS, and then developed in the chromogenic solution (70 mg DAB in 110 ml Tris-HCl). The sections were stained with Harris hematoxylin and then mounted.

### Morphometric Analysis

Conventional morphometric analysis was performed with the reticle of 100 points and 50 straight coupled to the microscope eyepiece (E200MV, Nikon Corporation, Tokyo, Japan) (Weibel, 2010). The total area of the reticulum was 10^4^ μm^2^, which was fixed at the base of the epithelium and the positive cells were quantified. The positive cells at 1,000× magnification were counted in four airway fields, in at least three airways, and 10 random fields in the alveolar septa per animal. Based on the number of points that coincided with the positive cells within the lattice divided by the number of points coincident with the peribronchial area for the airways or alveolar walls (Leick-Maldonado et al., 2004; Camargo et al., 2018).

### Image Analysis

Collagen fibers, decorin, fibronectin, biglycan, and isoprostane PGF-2α content were captured using a Leica DM2500 microscope (Leica Microsystems, Wetzlar, Germany) with a coupled camera that sent the images to a computer. Four airways and 10 fields per alveolar septa were quantified per animal. Images were analyzed using Image-Pro Plus 4.5 software (NIH, Bethesda, MD, United States). This software was set with a threshold for the color tones considered to be positive areas and automatically quantified these in the previously determined areas. The volume fractions of these markers were expressed as percentages of a total area (Righetti et al., 2014; Camargo et al., 2018).

### Gene Expression Analysis

The gene expression levels of IL-17, arginase, and VAChT were evaluated using RT-PCR (real-time polymerase chain reaction) (Pinheiro et al., 2015; Camargo et al., 2018). The primer sequences and annealing temperatures were: *GAPDH* (5′–3′ sense: CCACCACCCTGTTGCTGTAG; 5′–3′ antisense: CTTGGGCTACACTGAGGACC; 60°C; NM_008084), *VAChT* (5′–3′ sense: CCCTTTTGATGGCTGTG; 5′–3′ antisense: GGGCTAGGGTACTCATTAGA; 60°C; NM_10167164), *IL-17* (5′–3′ sense: TGAAGGTCAACCTCAAAGTCT; 5′–3′ antisense: GAGGGATATCTATCAGGGTCTTCAT) and *ARG-1* (5′–3′ sense GCACTCATGGAAGTACACGAGGAC, 5′–3′ antisense: CCAACCCAGTGATCTTGACTGA). The results were represented in cycle number (Ct) at which logarithmic PCR plots cross a calculated threshold line and used to determine ΔCt values [ΔCt = (Ct of the target gene) - (Ct of the house-keeping gene)]. The results were expressed as arbitrary units using the transformation: Expression = 1000 × (2^−Δct^) arbitrary units (AUs).

### Statistical Analysis

Scientific Graphing Software SigmaPlot^®^ Version 11.0 was used for all statistical analyses. We used one-way analysis of variance (ANOVA) followed by the Holm–Sidak method for multiple comparisons to evaluate the differences between groups. These results were expressed as means ± standard errors and all data are represented as the means ± standard deviation. For all analyses, *p* < 0.05 was considered statistically significant.

## Results

There were no differences among the control groups SAL, SAL-RHOi, SAL-anti-IL17, and SAL-RHOi-anti-IL17 in any of the evaluations performed. To facilitate visualization, the results of only one of the controls is shown in the graphs.

### Hyper-Responsiveness

**FIGURE 3:**
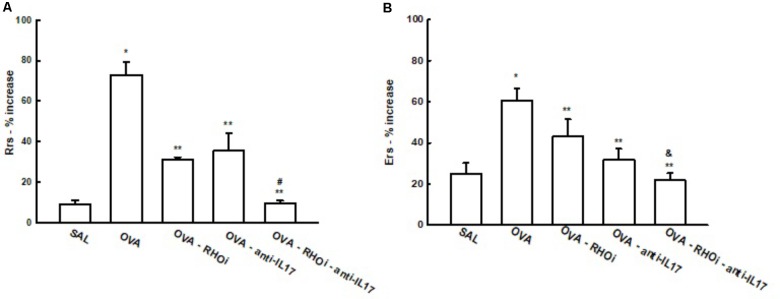
Effects of anti-IL17 and ROCK inhibitor treatment on hyper-responsiveness. **(A)** Percentage of maximal increase in respiratory system resistance (% Rrs) after challenge with methacholine, **(B)** percentage of maximal increase in elastance of the respiratory system (% Ers) after challenge with methacholine. ^∗^*p* < 0.05 compared to the SAL group;^∗∗^*p* < 0.05 compared to the OVA group; ^#^*p* < 0.05 compared to OVA-RHOi and OVA-anti-IL17; ^&^*p* < 0.05 compared to OVA-RHOi.

There was a significant increase of % Rrs in the OVA group after challenge with methacholine (73.0 ± 6.1%) compared to the control group (SAL: 9.137 ± 1.8%) (*p* < 0.05). Treatment of the sensitized animals with the ROCK inhibitor, anti-IL17, and the combination of the two attenuated this response (OVA-RHOi: 31.3 ± 0.9%, OVA-anti-IL17: 35.3 ± 8.5%, and OVA-RHOi-anti-IL17: 9.4 ± 1.4%), compared to the OVA group (*p* < 0.05). There was a decrease of % Rrs in the combination of the two treatments compared to the isolated treatments (*p* < 0.05) (**Figure 3**).

There was also an increase of % Ers in the OVA group (60.7 ± 5.7%) compared to the SAL control group (25.0 ± 5.1%) (*p* < 0.05). The treated groups that were OVA-RHOi (48.0 ± 8.2%), OVA-anti-IL17 (31.6 ± 5.5%) and OVA-RHOi-anti-IL17 (22.0 ± 3.3%) presented a decrease compared to the OVA group (*p* < 0.05). There was attenuation of % Ers when the combination of the two treatments was performed compared to OVA-RHOi group (*p* < 0.05).

**FIGURE 4:**
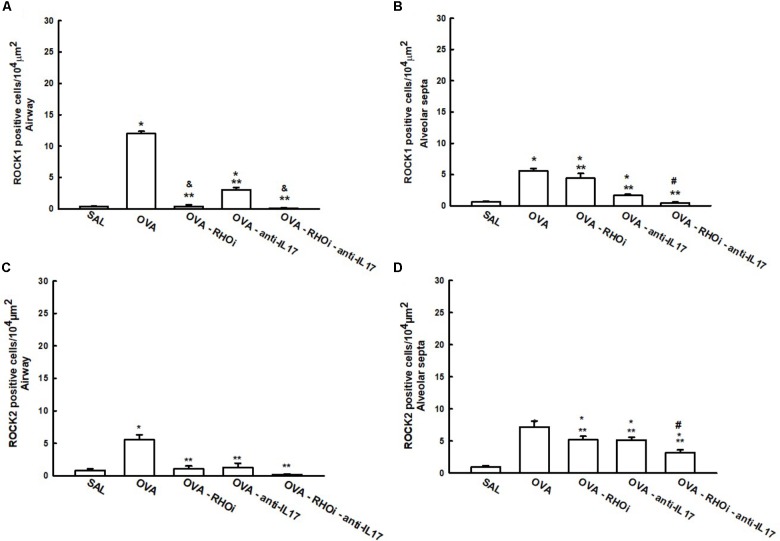
Effects of ROCK inhibitor and anti-IL17 in cells involved in hyper-responsiveness. **(A)** ROCK1 in the airway, **(B)** ROCK1 in the alveolar septa, **(C)** ROCK2 in the airway, and **(D)** ROCK2 in the alveolar septa, levels expressed as numbers of positive cells/10^4^ μm^2^. ^∗^*p* < 0.05 compared to the SAL group; ^∗∗^*p* < 0.05 compared to the OVA group; ^#^*p* < 0.05 compared to OVA-RHOi and OVA-anti-IL17 groups; & *p* < 0.05 compared to the OVA-anti-IL17 group.

ROCK1 and ROCK2 positive cells in the airways and alveolar septa are shown in **Figure 4**. There was an increase of ROCK1 and ROCK2 positive cells in the airways and alveolar septa in the OVA group compared to the SAL group (*p* < 0.05). ROCK inhibitor, anti-IL17 and the combination of the two treatments reduced the number of ROCK1 and ROCK2 positive cells in the airways and alveolar septa compared to OVA group (*p* < 0.05). An attenuation of the ROCK1 positive airway cells in the ROCK inhibitor-treated group and in the group with the combination of the ROCK inhibitor and anti-IL17 treatments compared to the OVA-anti-IL17 group (*p* < 0.05) It is also noted that the responses in the alveolar septa have potentiated the attenuation of the number of ROCK1 and ROCK2 positive cells in the groups treated with the combination of ROCK inhibitor and anti-IL17 (*p* < 0.05).

### Anti-IL-17 and ROCK Inhibitor on Inflammation

**FIGURE 5:**
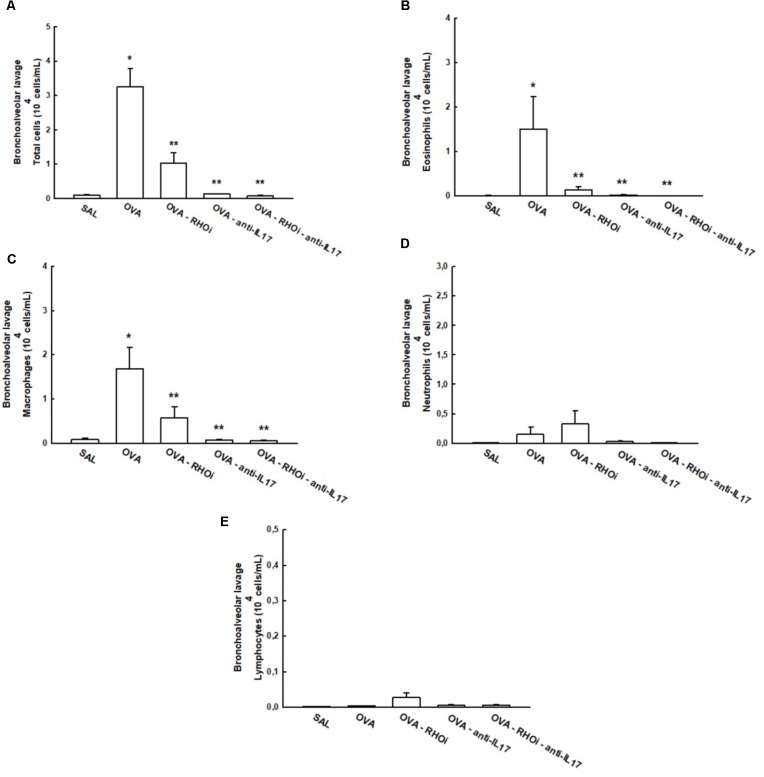
Effects of anti-IL17 and ROCK inhibitor treatment on BALF **(A)** Total cells, **(B)** cell differential for eosinophils, **(C)** cell differential for macrophages, **(D)** cell differential for neutrophils, **(E)** cell differential for lymphocytes. ^∗^*p* < 0.05 compared to SAL;^∗∗^*p* < 0.05 compared to OVA.

The total and differential cell counts in the BALF are shown in **Figure 5**. The OVA group (3.2 ± 0.5 10^4^cells/mL) showed an increase in the number of total cells compared to the SAL group (0.08 ± 0.02 10^4^cells/mL) (*p* < 0.05). Treatment of sensitized animals with ROCK inhibitor (OVA-RHOi: 1.01 ± 0.3 10^4^cells/mL), with anti-IL17 (0.1 ± 0.006 10^4^cells/mL) and the combination of the two (OVA-RHOi-anti-IL17: 0.06 ± 0.01 10^4^cells/mL) attenuated the number of total cells compared to the OVA group (*p* < 0.05). There were no differences between the SAL groups and the treated groups.

There was a significant increase in the number of eosinophils in the OVA group (1.5 ± 0.7 10^4^cells/mL) compared to the control group (SAL: 0.001 ± 0.0004 10^4^cells/mL) (*p* < 0.05). The treatment of sensitized animals with ROCK inhibitor (OVA-RHOi: 0.1 ± 0.07 10^4^cells/mL), with anti-IL17 (OVA-anti-IL17: 0.01 ± 0.007 10^4^cells/mL), and the combination of the two (OVA-RHOi-anti-IL17: 0.008 ± 0.003 10^4^cells/mL) decreased the number of eosinophils compared to the OVA group (*p* < 0.05). There were no differences between the control group and the treated groups. There was an increase in the number of macrophages in the OVA group (1.6 ± 0.4 10^4^cells/mL) compared to the SAL group (0.08 ± 0.02 10^4^cells/mL) (*p* < 0.05). Treatment of sensitized animals with ROCK inhibitor (OVA-RHOi: 0.5 ± 0.2 10^4^cells/mL), with anti-IL17 (OVA-anti-IL17: 0.07 ± 0.01 10^4^cells/mL), and the combination of the two treatments (OVA-RHOi-anti-IL17: 0.05 ± 0.009 10^4^cells/mL) reduced the number of macrophages compared to the OVA group (*p* < 0.05). There were no differences between the control group and the treated groups. There were also no differences among groups in terms of the number of the neutrophils and lymphocytes in the BALF.

Inflammatory markers CD4^+^, CD8^+^, IL-1β, IL-2, IL-4, IL-5, IL-6, IL-10, IL-13, IL-17, TNF-α, FOXP-3, dendritic cells, and NF-κB in the airway are shown in **Table 1** and the same inflammatory markers in the alveolar septa in **Table 2**. Considering the positive cell count of all of the inflammatory markers in the OVA group, there was an increase compared to the control group SAL (*p* < 0.05). All treated groups with association the anti-IL17 and ROCK inhibitor had a reduction in the number of positive cells compared to the OVA group (*p* < 0.05). There was a potential reduction in the number of IL-5 positive cells in the airway and the number of IL-4, IL-5, and FOXP3, positive cells in the alveolar septa in the group treated with the combination of anti-IL17 and ROCK inhibitors was potentiated compared to the individual treatments (*p* < 0.05 for all comparisons).

**Table 1:** Absolute values for inflammatory markers in the airway.

	Inflammatory markers	SAL	OVA	OVA-RHOi	OVA-anti-IL17	OVA-RHOi-anti-IL17
Airway	CD4^+^ (cells/10^4^μm^2^)	0.08 ± 0.08	7.17 ± 1.18^∗^	4.27 ± 0.93^∗,∗∗^	1.85 ± 0.53^∗∗^	2.41 ± 0.59^∗∗^
Airway	CD8^+^ (cells/10^4^μm^2^)	2.04 ± 1.15	23.75 ± 2.27^∗^	13.59 ± 2.51^∗,∗∗^	8.72 ± 3.21^∗∗^	4.6 ± 0.89^∗∗^, ^&^
Airway	IL-17 (cells/10^4^μm^2^)	0.57 ± 0.09	6.02 ± 0.22^∗^	1.89 ± 0.19^∗,∗∗^	0.96 ± 0.14^∗∗^, ^&^	0.84 ± 0.13^∗∗^, ^&^
Airway	IL-1β (cells/10^4^μm^2^)	2.35 ± 0.77	12.61 ± 2.18^∗^	4.66 ± 0.92^∗∗^	4.73 ± 0.94^∗∗^	3.52 ± 1.77^∗∗^
Airway	IL-2 (cells/10^4^μm^2^)	1.16 ± 0.17	4.99 ± 0.27^∗^	1.12 ± 0.43^∗∗^	1.71 ± 0.28^∗∗^	2.32 ± 0.86^∗∗^
Airway	IL-5 (cells/10^4^μm^2^)	0.9 ± 0.13	5.11 ± 0.28^∗^	2.09 ± 0.25^∗,∗∗^	1.59 ± 0.22^∗∗^	0.63 ± 0.12^∗∗^, ^$^
Airway	IL-6 (cells/10^4^μm^2^)	0.63 ± 0.11	5.79 ± 0.33^∗^	2.04 ± 0.57^∗^,^∗∗^	1.52 ± 0.14^∗∗^	1.1 ± 0.31^∗∗^
Airway	IL-10 (cells/10^4^μm^2^)	0.46 ± 0.36	9.44 ± 1.99^∗^	2.32 ± 0.72^∗∗^	3.84 ± 1.01^∗∗^	1.83 ± 0.61^∗∗^
Airway	IL-13 (cells/10^4^μm^2^)	0.09 ± 0.09	3.34 ± 1.32^∗^	1.13 ± 0.42^∗∗^	0.05 ± 0.05^∗∗^	1.03 ± 0.41^∗∗^
Airway	IL-4 (cells/10^4^μm^2^)	0.43 ± 0.31	4.11 ± 1.31^∗^	2.02 ± 0.65	1.3 ± 0.63	1.19 ± 0.44^∗∗^
Airway	TNF-α (cells/10^4^μm^2^)	2.04 ± 0.33	6.15 ± 0.64^∗^	3.26 ± 1.34	3.82 ± 0.73	1.58 ± 0.29^∗∗^
Airway	NF-κB (cells/10^4^μm^2^)	3.17 ± 0.82	6.6 ± 0.71^∗^	3.49 ± 0.86^∗∗^	3.09 ± 0.95^∗∗^	1.51 ± 0.74^∗∗^
Airway	FOXP3 (cells/10^4^μm^2^)	0.29 ± 0.22	7.24 ± 0.84^∗^	2.27 ± 0.98^∗∗^	3.79 ± 0.82^∗^,^∗∗^	0.7 ± 0.39^∗∗^
Airway	Dendritic cells (cells/10^4^μm^2^)	0.31 ± 0.31	10.5 ± 2.17^∗^	1.84 ± 0.53^∗∗^	3.23 ± 1.11^∗∗^	0.76 ± 0.27^∗∗^

*^∗^p < 0.05 compared to SAL group; ^∗∗^p < 0.05 compared to OVA group; ^$^p < 0.05 compared to OVA-RHOi and OVA-anti-IL17; ^#^p < 0.05 compared to OVA-anti-IL17; ^&^p < 0.05 compared to OVA-RHOi.*

**Table 2:** Absolute values for inflammatory markers in the alveolar septa.

	Inflammatory markers	SAL	OVA	OVA-RHOi	OVA-anti-IL17	OVA-RHOi-anti-IL17
Alveolar septa	CD4^+^ (cells/10^4^μm^2)^	0.82 ± 0.24	10.51 ± 1.17^∗^	2.97 ± 0.47^∗∗^	2.76 ± 0.47^∗∗^	2.3 ± 0.39^∗∗^
Alveolar septa	CD8^+^ (cells/10^4^μm^2^)	0.97 ± 0.35	14.75 ± 1.8^∗^	6.13 ± 0.77^∗, ∗∗^	5.71 ± 0.98^∗, ∗∗^	2.95 ± 0.71^∗∗^
Alveolar septa	IL-17 (cells/10^4^μm^2^)	0.32 ± 0.05	4.28 ± 0.18^∗^	1.4 ± 0.14^∗, ∗∗^	1.3 ± 0.39^∗, ∗∗^	1.09 ± 0.12^∗, ∗∗^
Alveolar septa	IL-1β (cells/10^4^μm^2^)	11.8 ± 1.37	20.25 ± 1.34^∗^	10.79 ± 0.85^∗∗^	14.85 ± 1.48^∗∗^	10.95 ± 1.24^∗∗^
Alveolar septa	IL-2 (cells/10^4^μm^2^)	0.928 ± 0.085	3.212 ± 0.106^∗^	1.084 ± 0.410^∗∗^	1.069 ± 0.117^∗∗^	0.997 ± 0.344^∗∗^
Alveolar septa	IL-5 (cells/10^4^μm^2^)	0.39 ± 0.07	5.07 ± 0.3^∗^	2.28 ± 0.3^∗, ∗∗^	1.97 ± 0.25^∗, ∗∗^	0.81 ± 0.163^∗∗, $^
Alveolar septa	IL-6 (cells/10^4^μm^2^)	0.915 ± 0.116	3.979 ± 0.224^∗^	2.085 ± 0.340^∗, ∗∗^	1.33 ± 0.16^∗∗^	1.33 ± 0.44^∗∗^
Alveolar septa	IL-10 (cells/10^4^μm^2^)	0.62 ± 0.19	5.86 ± 0.88^∗^	2.44 ± 0.54^∗∗^	3.48 ± 0.48^∗, ∗∗^	2.22 ± 0.36^∗, ∗∗^
Alveolar septa	IL-13 (cells/10^4^μm^2^)	0.722 ± 0.414	4.690 ± 0.596^∗^	1.150 ± 0.253^∗∗^	0.324 ± 0.159^∗∗^	0.853 ± 0.199^∗∗^
Alveolar septa	IL-4 (cells/10^4^μm^2^)	0.838 ± 0.290	5.227 ± 0.602^∗^	2.970 ± 0.494^∗, ∗∗, #^	5.512 ± 0.947^∗^	1.482 ± 0.295^∗∗, $^
Alveolar septa	TNF-α (cells/10^4^μm^2^)	1.182 ± 0.376	5.438 ± 0.543^∗^	3.401 ± 0.531^∗∗∗^	1.364 ± 0.278^∗∗, &^	1.056 ± 0.246^∗∗, &^
Alveolar septa	NF-κB (cells/10^4^μm^2^)	12.992 ± 1.346	23.893 ± 2.616^∗^	12.15 ± 1.08^∗∗^	7.587 ± 1.089^∗, ∗∗^	11.48 ± 0.75^∗∗^
Alveolar septa	FOXP3 (cells/10^4^μm^2^)	2.222 ± 0.285	7.758 ± 0.928^∗^	4.02 ± 0.7^∗∗^	4.449 ± 0.579^∗, ∗∗^	1.778 ± 0.304^∗∗, $^
Alveolar septa	Dendritic cells (cells/10^4^μm^2^)	1.139 ± 0.301	8.406 ± 1.387^∗^	2.760 ± 0.432^∗∗^	1.868 ± 0.458^∗∗^	0.65 ± 0.2^∗∗^

*^∗^p < 0.05 compared to SAL group; ^∗∗^p < 0.05 compared to OVA group; ^$^p < 0.05 compared to OVA-RHOi and OVA-anti-IL17; ^#^p < 0.05 compared to OVA-anti-IL17; ^&^p < 0.05 compared to OVA-RHOi.*

### Gene and Cell Expression of IL-17 in Inflammation

**FIGURE 6:**
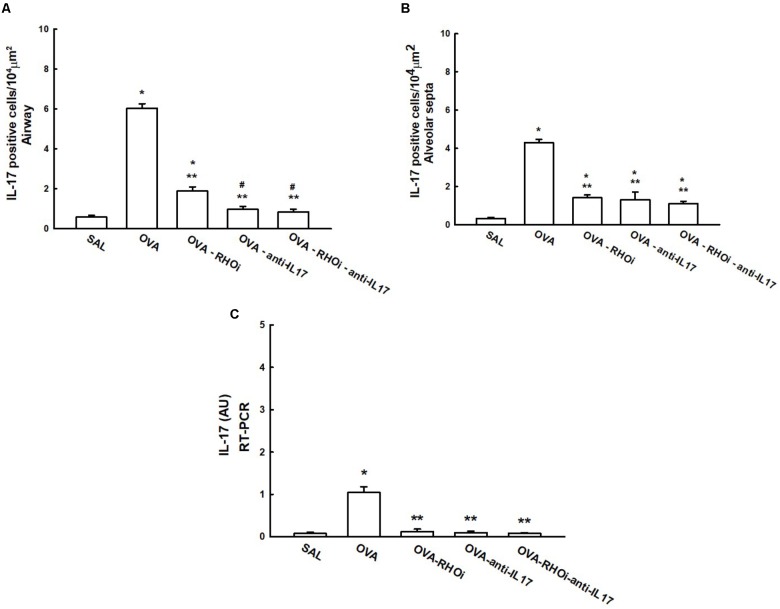
Effects of anti-IL17 and the ROCK inhibitor on IL-17 gene expression and IL-17 positive cell number. **(A)** Number of IL-17 positive cells in the airways, **(B)** number of IL-17 positive cells in the alveolar septa, and **(C)** the levels of IL-17 mRNA assessed by RT-PCR (AU). ^∗^*p* < 0.05 compared to the SAL group;^∗∗^*p* < 0.05 compared to the OVA group; ^#^*p* < 0.05 compared to OVA-RHOi group.

The number of IL-17 positive cells and the IL-17 gene expression data are shown in **Figure 6**. Evaluation of the number of IL-17 positive cells in the airways is shown in **Figure 6A**, there was an increase in the number of positive airway cells in the OVA group compared to the SAL group (*p* < 0.05). The evaluation of the number of IL-17 positive cells in the alveolar septa is shown in **Figure 6B**, there was an increase in the number of positive cells in the OVA group compared to the SAL group (*p* < 0.05). In the airways and alveolar septa, groups that received the ROCK inhibitor and anti-IL17 (OVA-RHOi, OVA-anti-IL17, and OVA-RHOi-anti-IL17) had attenuated inflammatory markers compared to the OVA group (*p* < 0.05). The gene expression results showed patterns similar to the morphometric analyses, as shown in **Figure 6C**.

### Extracellular Matrix Remodeling

The results of MMP-9, MMP-12, TIMP-1, and TGF-β positive cells and the volume fraction of collagen fibers, decorin, fibronectin, and biglycan in the airways are shown in **Table 3**, and alveolar septa are shown in **Table 4**. Extracellular matrix remodeling was increased in the OVA group compared to the SAL group (*p* < 0.05). There was a decrease in all markers in the OVA-anti-IL17, OVA-RHOi, and OVA-RHOi-anti-IL17 groups compared to the OVA group (*p* < 0.05). There was potentiation of the reduction of TGF-β positive cells in alveolar septa in the OVA-RHOi-anti-IL17 compared to the individual treatments (*p* < 0.05).

**Table 3:** Absolute values for remodeling markers in the airway.

	Remodeling markers	SAL	OVA	OVA-RHOi	OVA-anti-IL17	OVA-RHOi-anti-IL17
Airway	MMP-9 (cells/10^4^μm^2)^	2.4 ± 0.75	7.25 ± 1.79^∗^	3.49 ± 0.82^∗∗^	2.36 ± 0.72^∗∗^	1.37 ± 0.46^∗∗^
Airway	MMP-12 (cells/10^4^μm^2^)	0.51 ± 0.35	9.78 ± 0.81^∗^	0.51 ± 0.3^∗∗^	2.08 ± 0.84^∗∗^	2.35 ± 0.88^∗∗^
Airway	TIMP-l (cells/10^4^μm^2^)	0.21 ± 0.21	6.11 ± 1.24^∗^	1.57 ± 0.44^∗∗^	2.94 ± 1.01^∗∗^	1.66 ± 0.72^∗∗^
Airway	TGF-β (cells/10^4^μm^2^)	2.52 ± 0.91	12.01 ± 2.28^∗^	4.18 ± 1.06^∗∗^	2.84 ± 0.84^∗∗^	0.84 ± 0.41^∗∗^
Airway	Collagen fibers (%)	5.78 ± 0.73	16.48 ± 0.78^∗^	3.72 ± 0.44^∗∗^	3.45 ± 0.66^∗∗^	5.82 ± 0.48^∗∗^
Airway	Decorin (%)	0.41 ± 0.12	5.53 ± 2.53^∗^	0.58 ± 0.19^∗∗^	1.54 ± 0.31^∗∗^	1.55 ± 0.23^∗∗^
Airway	Fibronectin (%)	0.45 ± 0.08	1.0 ± 0.18^∗^	0.21 ± 0.04^∗∗, #^	0.98 ± 0.23^∗^	0.28 ± 0.05^∗∗, #^
Airway	Biglycan (%)	0.48 ± 0.09	1.32 ± 0.09^∗^	0.22 ± 0.05^∗∗^	0.6 ± 0.11^∗∗, &^	0.75 ± 0.09^∗∗, &^

*^∗^p < 0.05 compared to SAL group; ^∗∗^p < 0.05 compared to OVA group; ^#^p < 0.05 compared to OVA-anti-IL17; ^&^p < 0.05 compared to OVA-RHOi.*

**Table 4:** Absolute values for remodeling markers in the alveolar septa.

	Remodeling markers	SAL	OVA	OVA-RHOi	OVA-anti-IL17	OVA-RHOi-anti-IL17
Alveolar septa	MMP-9 (cells/10^4^μm^2)^	11.4 ± 1.25	19.78 ± 2.01^∗^	13.68 ± 1.08^∗∗^	8.3 ± 0.7^∗∗, &^	11.28 ± 0.8^∗∗^
Alveolar septa	MMP-12 (cells/10^4^μm^2^)	2.62 ± 0.55	7.39 ± 1.08^∗^	4.7 ± 0.71^∗∗^	4.16 ± 0.6^∗∗^	2.83 ± 0.43^∗∗^
Alveolar septa	TIMP-1 (cells/10^4^μm^2^)	0.83 ± 0.2	8.72 ± 0.85^∗^	5.65 ± 0.61^∗, ∗∗^	4.95 ± 0.61^∗, ∗∗^	4.75 ± 0.72^∗, ∗∗^
Alveolar septa	TGF-β (cells/10^4^μm^2^)	3.8 ± 0.7	14.59 ± 1.7^∗^	11.16 ± 0.96^∗, ∗∗^	7.95 ± 1.0^∗, ∗∗^	2.62 ± 0.48^∗∗, $^
Alveolar septa	Collagen fibers (%)	6.89 ± 0.5	12.43 ± 0.7^∗^	6.08 ± 0.35^∗∗, #^	4.39 ± 0.29^∗, ∗∗^	7.06 ± 0.38^∗, ∗∗, #^
Alveolar septa	Decorin (%)	5.57 ± 0.7	13.81 ± 1.05^∗^	5.28 ± 0.57^∗∗^	4.03 ± 0.38^∗∗^	5.06 ± 0.5^∗∗^
Alveolar septa	Fibronectin (%)	1.38 ± 0.09	3.02 ± 1.29^∗^	0.28 ± 0.04^∗, ∗∗, #^	1.23 ± 0.26^∗∗^	0.56 ± 0.07^∗, ∗∗^
Alveolar septa	Biglycan (%)	1.2 ± 0.11	3.64 ± 0.29^∗^	0.18 ± 0.05^∗, ∗∗^	1.54 ± 0.15^∗∗, &^	3.0 ± 0.26^∗, ∗∗, &^

*^∗^p < 0.05 compared to SAL group; ^∗∗^p < 0.05 compared to OVA group; ^$^p < 0.05 compared to OVA-RHOi and OVA-anti-IL17; ^#^p < 0.05 compared to OVA-anti-IL17; ^&^p < 0.05 compared to OVA-RHOi.*

### Oxidative Stress

**FIGURE 7:**
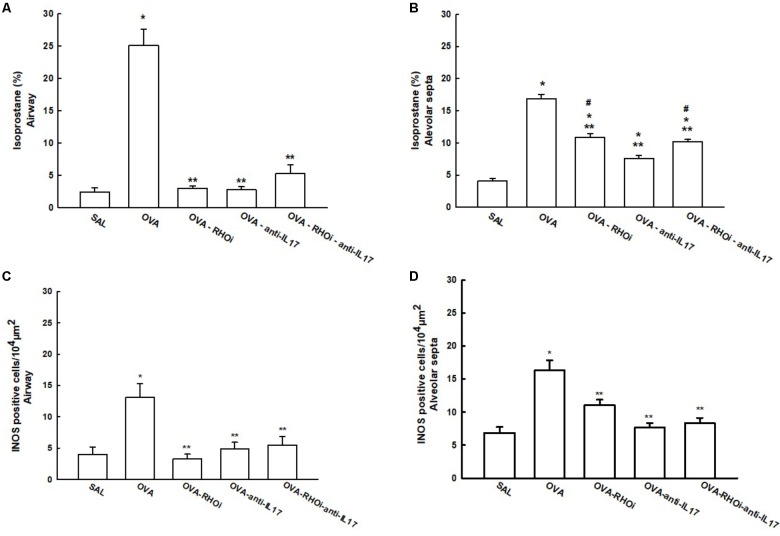
Effects of anti-IL17 and the ROCK inhibitor on oxidative stress. **(A,B)** 8-iso-PGF2α volume fraction in the airway and alveolar septa, respectively, and **(C,D)** iNOS-positive cells in the airway and alveolar septa, respectively. The results are expressed as positive cells/10^4^ μm^2^ and the volume fraction is expressed as percentages of total area (%). ^∗^*p* < 0.05 compared to the SAL group; ^∗∗^*p* < 0.05 compared to the OVA group; ^#^*p* < 0.05 compared to the OVA-anti-IL17 group.

The content of isoprostane PGF-2α and iNOS positive cells in the airways and alveolar septa are shown in **Figure 7**. There was an increase in the percentage of isoprostane PGF-2α in the OVA group in the airways (25.1 ± 2.5%) and alveolar septa (16.896 ± 0.6%) compared to the SAL group in the airway (2.3 ± 0.6%) and alveolar septa (4.09 ± 0.3%) (*p* < 0.05). Treatment of the animals with the ROCK inhibitor (OVA-RHOi: 2.9 ± 0.3%), with anti-IL17 (OVA-anti-IL17: 2.7 ± 0.4%), and the combination of the two treatments (OVA-RHOi-anti-IL17: 5.2 ± 1.3%) reduced isoprostane PGF-2α compared to the OVA group in the airway (*p* < 0.05). There were no differences between the treated groups and the SAL control group in the airways. In the alveolar septa, ROCK inhibitor treatment (OVA-RHOi: 10.8 ± 0.5%), with anti-IL17 (OVA-anti-IL17: 7.5 ± 0.4%), and the combination of the two treatments (OVA-RHOi-anti-IL17: 10.19 ± 0.3%) decreased isoprostane PGF-2α compared to the OVA group (*p* < 0.05). There was a reduction of isoprostane PGF-2α in the alveolar septa in the OVA-anti-IL17 group compared to the OVA-RHOi and OVA-RHOi-anti-IL17 groups (*p* < 0.05).

There was an increase in the number of iNOS positive cells in the OVA group in the airways (13.08 ± 2.1 cells/10^4^ μm^2^) and alveolar septa (16.30 ± 1.5 cells/10^4^ μm^2^) compared to the SAL group in the airways (4.01 ± 1.1 cells/10^4^ μm^2^) and alveolar septa (6.86 ± 0.8 cells/10^4^ μm^2^). There were no differences among the treated groups in the airways and alveolar septa compared to the SAL group. In the airway, treatment with the ROCK inhibitor (OVA-RHOi: 3.24 ± 0.7 cells/10^4^ μm^2^), anti-IL17 (OVA-anti-IL17: 4.8 ± 1.4 cells/10^4^ μm^2^), and the combination of the two (OVA-RHOi-anti-IL17: 5.4 ± 1.3 cells/10^4^ μm^2^) significantly reduced the number of positive cells compared to the OVA group (*p* < 0.05). In the alveolar septa, the treatments had the same response (OVA-RHOi: 11.0 ± 0.0 cells/10^4^μm^2^), anti-IL17 (OVA-anti-IL17: 7.6 ± 0.6 cells/10^4^μm^2^), and the combination of the two treatments (OVA-RHOi-anti-IL17: 8.3 ± 0.8 cells/10^4^μm^2^) compared to the OVA group (*p* < 0.05).

### Inflammatory Mechanism

**FIGURE 8:**
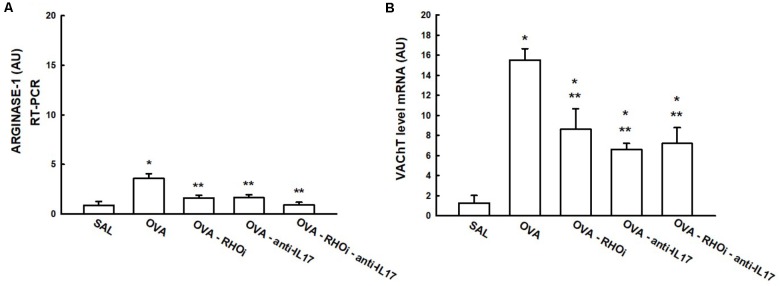
Effects of anti-IL17 and the ROCK inhibitor on arginase-1 **(A)** and VAChT **(B)** gene expression. The levels of mRNA in the lung were evaluated using RT-PCR (AU). ^∗^*p* < 0.05 compared to the SAL group; ^∗∗^*p* < 0.05 compared to the OVA group.

The gene expression of arginase-1 and VAChT in the lung is presented in **Figure 8**. In the VAChT gene expression data (**Figure 8B**), treatment with the ROCK inhibitor (OVA-RHOi: 8.6 ± 2.0 level mRNA AU), treatment with anti-IL17 (OVA-anti-IL17: 6.6 ± 0.6 level mRNA AU), and the combination of the two treatments (OVA-RHOi-anti IL-17: 7.2 ± 1.5 level mRNA AU) attenuated VAChT expression compared to the OVA group (*p* < 0.05). From the arginase-1 expression data (**Figure 8A**), treatment with ROCK inhibitors (OVA-RHOi: 1.6 ± 0.2 level mRNA AU) with anti-IL17 (OVA-anti-IL17: 1.6 ± 0.2 level mRNA AU), and the combination of the two treatments (OVA-RHOi-anti-IL17: 0.9 ± 0.2 level mRNA AU) significantly reduced arginase expression relative to the OVA group (*p* < 0.05). In the evaluation of the PCR for IL-17 and arginase, there were no differences between the OVA-RHOi, OVA-anti-IL17, and OVA-RHOi-anti-IL17 groups and the SAL control group.

### Qualitative Analysis

**FIGURE 9:**
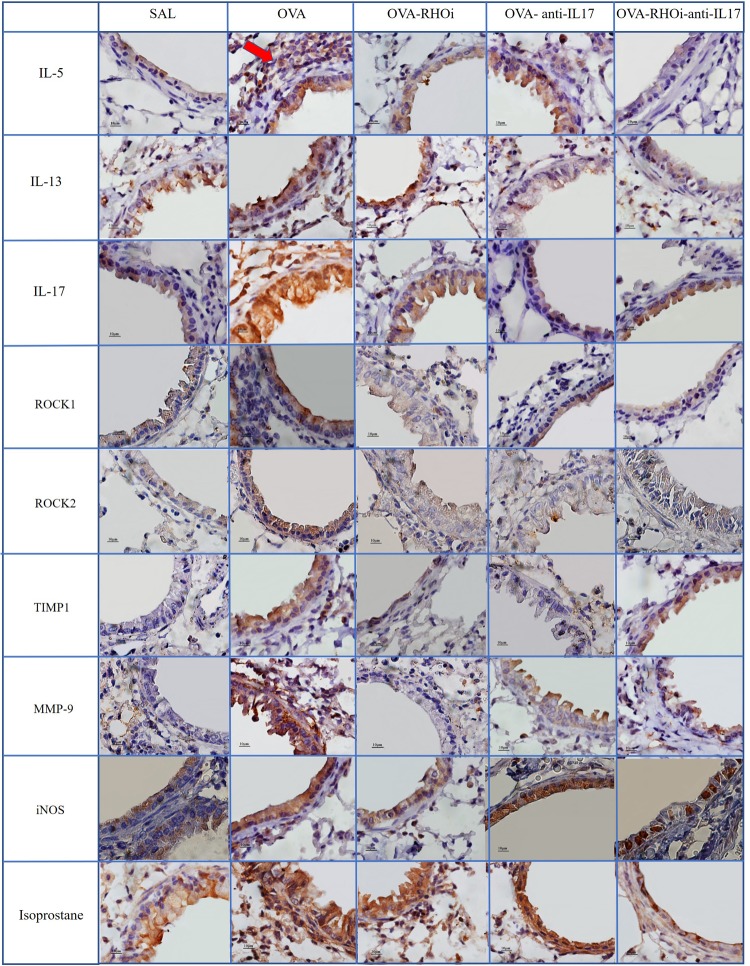
Inflammatory, extracellular matrix remodeling and oxidative stress markers in airways: Photomicrographs of IL-5, IL-13, IL-17, ROCK1, ROCK2, TIMP-1, MMP-9, iNOS, and isoprostane immunohistochemical stain in the airways. 1,000× magnification. All experimental groups are represented: SAL, OVA, OVA-RHOi, OVA-antiIL17, and OVA-RHOi-anti-IL17 groups. The red arrow indicate de positive cells.

**FIGURE 10:**
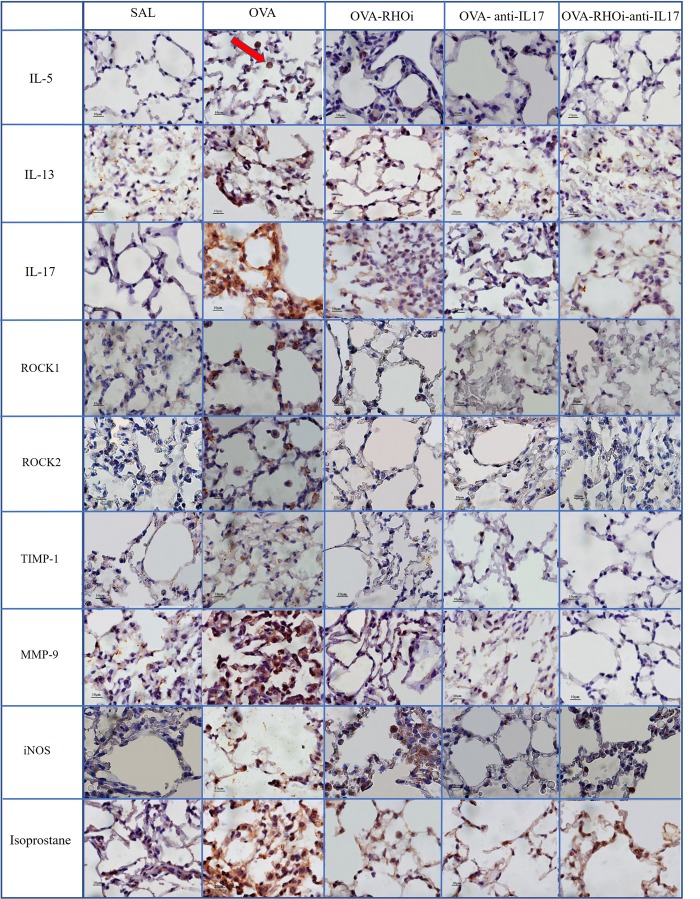
Inflammatory, extracellular matrix remodeling and oxidative stress markers in alveolar septa: Photomicrographs of IL-5, IL-13, IL-17, ROCK1, ROCK2, TIMP-1, MMP-9, iNOS and isoprostane immunohistochemical stain in lung tissue. 1000× magnification. All experimental groups are represented: SAL, OVA, OVA-RHOi, OVA-antiIL17 and OVA-RHOi-anti-IL17 groups. The red arrow indicate de positive cells.

Representative photomicrographs are presented in **Figures 9**, **10**, and illustrate the inflammatory processes, extracellular matrix remodeling and oxidative stress in the airways and alveolar septa. **Figure 9** exhibits the airway and **Figure 10** illustrates the alveolar septa. Inflammatory processes are illustrated by IL-5, IL-13, IL-17; the characteristics of extracellular matrix remodeling by TIMP-1, MMP-9, and markers of oxidative stress by iNOS and isoprostane. For understanding of the mechanisms of mechanical alterations we also assessed the ROCK1 and ROCK2 expressions. ROCK inhibitor and anti-IL17 treatment reduced the above changes in airways and alveolar septa, which can be observed in the representative photomicrographs. We delineate the positive cells to exemplify by arrows. All the microphotographs were taken 1000x magnification.

## Discussion

The present study demonstrated that animals treated with an IL-17 neutralizing antibody or with the ROCK inhibitor alone controlled the hyper-responsiveness to methacholine in both the resistance and the elastance of the respiratory system compared to the sensitized and untreated animals. In addition, there was attenuation of the number of total cells in the BALF, including eosinophils and macrophages. In the treated groups, there was also a reduction in the number of CD4^+^, CD8^+^, IL-1β, IL-2, IL-17, IL-5, IL-6, IL-4, IL-10, IL-13, NF-κB, TNF-α, FOXP3, dendritic cells, MMP-9, MMP-1, TGF-B, ROCK1 and ROCK2 positive cells. We noted a reduction in IL-17, VAChT, and arginase gene expression and the amount of isoprostane PGF-2α, decorin, biglycan, fibronectin, and collagen fibers was also decreased.

When the treatments were combined, they potentiated a reduction of the responses of resistance of respiratory system to methacholine. In the airway, there was also a potentiation of the reduction of IL-5 positive cells; and in the alveolar septa, IL-5, FOXP3, TGF-β, ROCK1, and ROCK2 positive cells.

The evaluation of resistance of respiratory system showed that both individual treatments attenuated the maximal response to methacholine, but the association of both treatments potentiated this response. In these regard, the responses of Rrs are associated with the inflammatory response (Rizzo et al., 2002). Our study showed that only the IL-5 expression in airway walls was significantly reduced in the OVA-RHOi-anti-IL17 compared to OVA-RHOi and OVA-anti-IL17. This data corroborates the importance of IL-5 in the modulation of airway hyper-responsiveness.

On the other hand, the maximal response of elastance was associated to alterations in alveolar septa. FOXP-3, IL-5, TGF-β, ROCK1, and ROCK2 were significantly reduced in alveolar septa compared to OVA-RHOi and OVA-anti-IL17.

Schaafsma et al. (2008) verified the ability of pre-treatment with Y-27632 to decrease the acute and late allergen-induced responses to ovalbumin. (Schaafsma et al., 2004) also showed a protective effect against the development of hyper-responsiveness in the airways, suggesting that the inhibitor confers a relaxation effect, and is, therefore, promising for the treatment of airway diseases. In a study by Righetti et al. (2014), the inhibition of ROCK in animals chronically exposed to ovalbumin attenuated the hyper-responsiveness of the distal parenchyma.

The use of Y-27632 in mice sensitized with ovalbumin attenuated the airway contractility caused by methacholine (Witzenrath et al., 2008). Possa et al. (2012) also demonstrated in an asthma model with chronic allergic inflammation that hyper-responsiveness to an antigen challenge was reduced by the use of Y-27632 in sensitized animals. Pigati et al. (2015) demonstrated that treatment with the Y-27632 inhibitor or corticosteroid treatment reduced the airway resistance response to ovalbumin in the sensitized and untreated group. However, there was no potentiation of this response in the group that received the combination of the two treatments. Taki et al. (2007) demonstrated that treatment with Fasudil suppressed hyper-responsiveness to methacholine induced by challenges of ovalbumin. In an *in vitro* study in mice, Kudo et al. (2012) found that anti-IL17 can attenuate the contractile response of smooth muscle cells in the airways. Manni et al. (2016) demonstrated that IL-17A contributes independently to airway hyper-responsiveness in an experimental model of mixed steroid-resistant Th2/Th17 asthma.

However, there are no studies that evaluate the effects of the combination of the two treatments on bronchial hyper-responsiveness.

In the present study, treatment with the ROCK inhibitor with and without anti-IL17 decreased the number of ROCK1 and ROCK2 positive cells in relation to the sensitized control group. We also illustrated in the panel of photomicrographs this reduction (**Figures 9**, **10**). There was potentiation of the control of this response in the alveolar septa in the group in which ROCK inhibitor treatment was combined with anti-IL17 treatment. These data reinforce the efficacy of the ROCK inhibitor, which not only blocks the action of ROCK but also reduces the expression of the enzyme itself.

One pathway that is related to the response and activation of RhoA is the heterotrimeric guanine nucleotide-binding proteins (G proteins), signal transducers that connect receptors to effectors and thus to intracellular signaling pathways (Neves et al., 2002). In fact, Gαq/11 family has been shown to activate RhoA (Sánchez-Fernández et al., 2014). Gq-dependent signaling rather than a receptor phosphorylation-dependent pathway was primarily responsible for airway constriction *ex vivo* and *in vivo*. Increasing evidence indicates that ligands for G protein-coupled receptors can also activate NF-κB (Matthey et al., 2017).

Camargo et al. (2018) showed in an asthma model that anti-IL17 controlled the ROCK1 and ROCK2 expression an also NF-κB expression.

In addition, Pantano et al. (2008) also demonstrated that epithelial activation of NF-κB increased the levels of IL17. However, the mechanisms involved were also a matter of controversy since Liu et al. (2015) indicated that Gαq negatively controls the differentiation of Th17 cells.

In regards to eosinophilic infiltration, treatment with Y-27632, anti-IL17, or the combination of the two attenuated this response in the sensitized animals. In the three treatment groups, there were no differences in eosinophil values compared to the saline control group. Adachi et al. (2001) evaluated eosinophils isolated from human peripheral blood following administration of Y-27632 and demonstrated that the use of this inhibitor reduced eosinophilia due to decreased eotaxin signaling. Pre-treatment with this inhibitor also reduced the number of eosinophils in the BALF of mice sensitized with ovalbumin (Henry et al., 2005).

After administering Fasudil to mice challenged with ovalbumin, Taki et al. (2007) observed a reduction in the number of cells including eosinophils in the BALF in the airways and in the blood vessels. Both infiltration of inflammatory cells and goblet cell hyperplasia were attenuated in Fasudil-treated animals. Possa et al. (2012) also demonstrated a decrease in the number of eosinophils in the airways in an asthma model treated with Y-27632. Pigati et al. (2015) also found a reduced number of eosinophils after Y-27632 treatment associated or not with the corticosteroid in an experimental model of asthma. In experimental models of asthma with IL-17A deficiencies, there was a reduction in the number of neutrophils and eosinophils in the airways (Schnyder-Candrian et al., 2006; Wakashin et al., 2008). Results from the present study are in agreement with previous studies, which, taken together, strongly support that treatment with anti-IL17A inhibits eosinophilic inflammation (Song et al., 2008; Lajoie et al., 2010; Willis et al., 2015).

Again, we could not find any studies on the combination of a ROCK inhibitor and anti-IL17 in experimental models of asthma that evaluate the eosinophilic response.

Schaller et al. (2005) studied the role of the CD8^+^ cell inhibitor in sensitized mice and demonstrated that it decreases the hyper-responsiveness and IL-2, IL-5, and IL-13 cytokine levels. These data indicate that CD8^+^ cells also play an important role in the exacerbation of the allergic responses by modulating production of pro-inflammatory cytokines. In an experimental model of asthma, Wang and Wills-Karp (2011) demonstrated that CD4^+^/Th2 cells induce lung inflammation associated with IL-17 production in the chronic phase of the disease.

In this study, there were fewer IL-1β, IL-2, IL-4, IL-5, IL-6, IL-10, IL-13, IL-17, and TNF-α positive cells in the groups treated with ROCK inhibitor or anti IL-17 alone compared to the sensitized and untreated control groups. There was no potentiation of the response when the two treatments were combined, except in relation to the IL-5 positive cells in the airways and IL-5 expression in the alveolar septa. Possa et al. (2012) demonstrated that animals exposed to ovalbumin that received ROCK inhibitor treatment experienced a reduction in IL-13 positive cells in the airways compared to the sensitized control group. Pigati et al. (2015) found that ovalbumin-exposed animals treated with a ROCK inhibitor showed decreased numbers of IL-5 and IL-13 positive cells in the airways and lung tissue compared to the untreated control group. We also noted this reduction in the number of positive cells for inflammatory markers such as IL-5, IL-13, and IL-17 in the panel of photomicrographs (**Figures 9**, **10**).

Several studies showed that in models of intense inflammatory responses, IL-10 may be elevated as a contra regulatory mechanism. In a study using *S. japonicum*-infected or OVA sensitized asthma murine models, both *in vivo* and *in vitro* data proved that IL-10 has opposite regulation on Treg cells. In another words, the regulatory effects of IL-10 on immune responses can be bidirectional (Mo et al., 2008).

Some authors suggest a new subset of Th17 producing IL-10 cells that do not induce tissue inflammation and they contribute, actually, to inhibit autoimmune inflammation. Thus, Th17 producing IL-10 cells appears to act as a double-edged sword in the pathogenesis of various diseases. Several studies indicated that Th17 cells can *trans*-differentiate into a Th1 or Th2 cell phenotype, thus the plasticity of Th17 cells may be dependent on the dominant factor in the surrounding milieu (Zielinski et al., 2012; Wu et al., 2018). In addition, our findings are in agreement with Camargo et al. (2018) that also demonstrated the increase of IL-10 in the group of animals exposed to ovalbumin. The response of our study was similar to that of these authors. We noticed an increase in the number of IL-10 positive cells in the OVA group when compared to the control group, and a decrease of these cells in the treated groups when compared to the OVA group.

Evidence shows that TNF-α plays a critical role in the initiation and elevation of airway inflammation in patients with asthma (Berry et al., 2007; Brown et al., 2015). In this present study, ROCK inhibitor with anti-IL17 attenuated the number of TNF-α positive cells both in the airway and in the alveolar septa compared to the sensitized and untreated control groups. TNF-α induces complex signaling events in endothelial cells (ECs) that lead to transcription changes including increased expression of inflammatory genes. Mong et al. (2008) suggested that ROCK is involved in the endothelial response to TNF-α by increasing IL-6 production. In the treatment of rheumatoid arthritis, TNF-α and IL-17 have shown a synergistic effect in promoting the production of IL-6 and IL-8 (Fischer et al., 2015). We did not find studies evaluating the combination of treatments on the expression of these cytokines.

In terms of collagen fibers, the two treatments alone and in combination attenuated the response compared to the sensitized and untreated control groups. The most effective treatment was observed in the OVA-anti-IL17 group in the alveolar septa. Possa et al. (2012) showed similar results: treatment with the ROCK inhibitor in animals exposed to ovalbumin reduced the content of collagen fibers. The same results were found by Pigati et al. (2015), who also observed in an experimental model of asthma treated with ROCK inhibitor a reduction in collagen fiber content in the airways and alveolar septa compared to the OVA group (Witzenrath et al., 2008).

Regarding remodeling markers such as MMP-9 and TIMP-1, a previous studies presents similar results to ours (Pigati et al., 2015), which showed an increase in the number of MMP-9 and TIMP-1 positive cells in the airways and lung tissue of the OVA group compared to the SAL group. In an asthma model, Possa et al. (2012) demonstrated attenuation of the number of MMP-9 and TIMP-1 positive cells in animals receiving a ROCK inhibitor compared to untreated ovalbumin-exposed animals. Camargo et al. (2018) demonstrated a decrease in MMP-9 and TIMP-1 positive cells in animals exposed to ovalbumin and treated with anti-IL17. These same responses were found in the present study, showing a decrease in these markers of remodeling in the airways and alveolar septa in the animals treated with the ROCK inhibitor or anti-IL17, being also illustrated in the photomicrographs (**Figures 9**, **10**).

A study by Kolb et al. (2001) showed that *in vivo* only decorin and not biglycan interfered in the activity of TGF-b. Weitoft et al. (2014) speculated that increased amounts of decorin could regulate interfibrillar spacing by creating a stiffer collagen matrix that influenced lung elasticity while also having a protective effect by minimizing remodeling. Similar results to our are shown in previous studies, including findings using cell culture models that suggest that decorin and collagen I in combination work through the Rho/Rock pathway (Goetsch et al., 2011). Camargo et al. (2018) demonstrated in an experimental model that the anti-IL17 treated group had a decrease in the percentage of fibronectin, decorin, and biglycan in the pulmonary parenchyma compared to the group not treated with the antibody. A study by Weitoft et al. (2014) reports similar findings to ours. They found the expression of decorin was increased in the central airways and alveolar parenchyma of patients with uncontrolled asthma compared to patients with controlled asthma and controls groups. In the central airways and alveolar parenchyma, the percentage of biglycan area decreased in patients with controlled asthma compared to patients with uncontrolled asthma (Weitoft et al., 2014). In our study, the treated groups showed a decrease in the percentage of decorin in the airways and alveolar septa compared to the untreated group.

Peters et al. (2016) demonstrated that *in vitro* stimulation of mouse fibroblasts with IL-17A resulted in increased TGF-β release and production of collagen fibers. There are no *in vivo* studies evaluating collagen fibers in the airways and alveolar septa in anti-IL17 treated animal models of asthma. It should also be noted that the same was observed in studies with ROCK inhibitors and anti-IL17.

In our study, there was a decrease in the content of biglycan in all treated groups when compared to the OVA group. There was a decrease in the content of biglycan in the OVA-RHOi group when compared to the group that performed the association of treatments.

One point is that the tendency of collagen response in OVA-RHOi-anti-IL17 in the alveolar septa was similar to that observed concerning biglycan. It is well known that collagen fibers interact with proteoglycans such as biglycan in the extracellular matrix. Biglycan was one of the most abundant proteoglycans that have a binding site to collagen I (Robinson et al., 2017).

Biglycan are proteolytically secreted by circulating macrophages and engages TLR2/4. This initiates a pro-inflammatory cascade that converges on NF-κB and evokes the synthesis and development of mature IL-1β, release of TNF-α and IL-6. Biglycan in its soluble form acts as danger signal bridging the innate and adaptative immune systems. Monomeric biglycan interacts with extracellular matrix components, leading to sequestration of a potentially proinflammatory signal.

Biglycan induces secretion of mature IL-1β, a proinflammatory cytokine important both in acute and chronic inflammation (Nastase et al., 2012). This made us to think that perhaps IL-1β interleukin might be involved in this response. Study *in vitro* incubation with IL-1β reduced the calcium sensitivity and suppressed the activities of ROCK (Liang et al., 2013). Rock may play an important role in IL-1. Inhibiting ROCK activity suppressed IL-1 secretion (Banerjee and McGee, 2016).

In relation to IL-17, IL-1 family cytokines possess a potent adjuvant activity to promote both Th2 and Th17 cells. IL-1β may mediate the early introduction of naive CD4^+^ T cells toward the Th2 type. It may then redirect them to the Th17 type. Th17 cells express IL-1R and require IL-1 for the differentiation from naive T cells (Zielinski et al., 2012; Kobayashi et al., 2013). For this reason, we evaluated the number of IL-1-positive cells in airway and alveolar septa.

However, the responses were not similar to those related to the biglycan. So, we believe that more studies are still required to substantiate the response of biglycan and involved mechanisms when there is inhibition of ROCK and the use of anti-IL17 associated.

To investigate the oxidative stress response, we quantified the content of PGF-2α isoprostane. There was a reduction in the PGF-2α isoprostane content of the groups treated with the inhibitors alone and in combination compared to the sensitized and untreated control groups. This reduction of the PGF-2α isoprostane content can be observed in **Figures 9**, **10**.

Possa et al. (2012) also observed that animals treated with Y-27632 had less PGF-2α isoprostane. Pigati et al. (2015) demonstrated that the amount of isoprostane PGF-2α in the airways and alveolar septa was lower in animals treated with a ROCK inhibitor, corticosteroid, and both combined compared to the OVA control group. In addition, studies have indicated that ROCK may be involved in the airway hyper-reactivity response, noting that the increase generated by the administration of histamine and PGF-2α is reversed by Y-27632 (Schaafsma et al., 2004; Shiraki et al., 2009).

A study describing the increased expression of FOXP3 in patients with allergic asthma relative to non-allergic asthma and Treg cell suppressor capacity observed in both are similar to our findings (Raedler et al., 2015). In our study, we showed an increase in the expression of FOXP3 in the OVA group compared to the SAL group and this was attenuated upon treatment with anti-IL17 and the ROCK inhibitor. This attenuation was enhanced in the alveolar septa by the combination of the two treatments.

PCR evaluation of arginase expression documented its increase in the OVA group compared to the control group and the attenuation of this elevated expression in the treated groups.

Several authors have suggested the importance of the oxidative stress response and activation of NO-arginase pathways in the pathophysiology of asthma, based on a correlation between these responses and the severity of asthma symptoms associated with airflow limitation, hyper-reactivity, and airway remodeling (Brussino et al., 2010; Voynow and Kummarapurugu, 2011). Studies have reported an increase in arginase activity in both acute and chronic asthma models (Meurs et al., 2000; Maarsingh et al., 2008, 2009; Aristoteles et al., 2013). Aristoteles et al. (2013) demonstrated in an experimental model of chronic asthma that modulation of NF-κB contributed to the activation of arginase and iNOS pathways, leading to increased numbers of arginase and iNOS positive cells associated with constriction of the distal pulmonary parenchyma. However, no studies have yet investigated arginase expression in the presence of a combination of anti-IL17 and a ROCK inhibitor.

The cholinergic pathway has been described as an important modulator of the inflammatory response (Prado et al., 2011). Pinheiro et al. (2015) demonstrated that VAChT deficiency-induced airway inflammation with increased levels of TNF-α and IL-4. Mice with decreased levels of VAChT showed increased deposition of collagen fibers and elastic fibers in their airway walls, consistent with an increase in MMP-9 and TIMP-1 positive inflammatory cells. The evaluation of lung function *in vivo* showed hyperactivity of the airways to methacholine in VAChT-deficient mice; the authors concluded that an intact cholinergic pathway is necessary for maintaining the homeostasis of the lung (Pinheiro et al., 2015). In the present study, PCR evaluation revealed an increase in VAChT expression in the OVA group compared to the control group and a reduction in this expression in the treated groups compared to this OVA group.

In the present experimental model in which a chronic inflammatory process is clearly established, the increase in VAChT expression is likely due to an anti-inflammatory property of the cholinergic system. When the inflammatory process was attenuated by the inhibitory treatments, there was also a decrease in this response.

The present study had some limitations. Exist the limitation the use of experimental animal model, because we cannot directly extrapolate our findings to those expected in humans. However, our results supported the therapeutic importance of anti-IL17 and the ROCK inhibitor in the control of inflammation, remodeling of the extracellular matrix and activation of oxidative stress pathway.

We performed the evaluation of oxidative stress through the cellular expression of iNOS, PGF-2α isoprostane content and RT-PCR for arginase. We considered that this selection of oxidative stress markers were adequate since a high production of NO by iNOS activation is associated to peroxynitrite formation. It is well known the importance of this pathway in the modulation of ROCK/RhoA activation and modulation of airway constriction. For future studies we will consider the inclusion of more oxidative stress markers as suggested by the reviewer.

However, our study has strengths. We considered the importance of the evaluation of various inflammatory mediators and contractile markers in the presence of anti-IL-17 antibody, ROCK inhibitor, or in combination. We analyzed a large number of inflammatory markers for Th1, Th2, and Th17 cytokines. We also performed a detailed analysis of the remodeling process, including collagen fibers, MMP-9, MMP-12, TIMP-1, TGF-β, decorin, biglycan, and fibronectin.

## Conclusion

In this study, the ROCK inhibitor and anti-IL17 neutralizing antibody were shown to contribute to the control of T helper cell-mediated inflammation, hyper-responsiveness, pro-inflammatory response, remodeling, and oxidative stress in the airways and alveolar septa in a murine model of chronic allergic inflammation. Treatment with anti-IL17 combined with the ROCK inhibitor potentiated the effect of decreasing the percentage of resistance increase after challenge with methacholine, the attenuation of IL-5 positive cells in the airway, and the reduction of IL-5, TGF-β, FOXP3, ROCK1, and ROCK2 positive cells in the alveolar septa; therefore, this combinatorial approach represents a promising new therapeutic strategy.

## Ethics Statement

All BALB/c mice from the School of Medicine of the University of São Paulo were treated according to the Laboratory Animal Care and Use Guideline, published by the National Institutes of Health (NIH publication 85-23, revised in 1985). Ethics Committee on the Use of Animals of the School of Medicine of the University of São Paulo approved the all procedures in this study (Case No. 064/15).

## Author Contributions

TdS, IT, MM, CP, and RR conceived and designed the experiments. TdS, EL, BS-R, MA-V, RR, FdS, SF, CP, LC, MC, and LA performed the experiments. TdS, RR, CP, EL, and IT analyzed the data. RR, IT, MM, EL, and MA-V contributed reagents, materials, and analysis tools. TdS, IT, EL, and RR wrote the paper.

## Conflict of Interest Statement

The authors declare that the research was conducted in the absence of any commercial or financial relationships that could be construed as a potential conflict of interest.

## Abbreviations

8-iso-PGF2α: 8-isoprostane-PGF2α
BALF: bronchoalveolar lavage fluid
BSA: bovine serum albumin
Ers: respiratory elastance
IL: interleukin
iNOS: inducible nitric oxide synthase
MMP-9: metalloproteinase 9
MMP-12: metalloproteinase 12
NF: nuclear factor
ROCK: Rho-kinase
Rrs: respiratory resistance
TIMP1: tissue inhibitor of metalloproteinase-1
TNF-α: tumor necrosis factor-α.
